# Community Resource Uses and Ethiopian Wolf Conservation in Mount Abune Yosef

**DOI:** 10.1007/s00267-015-0529-6

**Published:** 2015-05-14

**Authors:** Girma Eshete, Girmay Tesfay, Hans Bauer, Zelealem Tefera Ashenafi, Hans de Iongh, Jorgelina Marino

**Affiliations:** Conservation Biology Department, Institute of Environmental Sciences, University of Leiden, P.O. Box 9518, 2300 RA Leiden, The Netherlands; North Wollo Zone Environmental Protection Department, P.O. Box 461, Woldia, Ethiopia; Ethiopian Wolf Conservation Programme, P.O. Box 215, Robe, Bale Ethiopia; College of Dryland Agriculture, Mekelle University, P.O. Box 231, Mekelle, Ethiopia; Wildlife Conservation Research Unit, Zoology Department, The Recanati-Kaplan Centre, University of Oxford, Tubney House, Abingdon, OX13 5QL UK; Frankfurt Zoological Society, P.O. Box 101426, Addis Ababa, Ethiopia

**Keywords:** Afroalpine ecosystem, Attitudes, Ethiopian highlands, Human-wildlife conflict, Natural resources

## Abstract

People who perceive economic benefits and enjoy unrestricted access to natural resources tend to support ecosystem conservation efforts. Our study explores whether this remains true in remnant patches of Afroalpine ecosystem in North Ethiopia, where communal land provides valuable natural resources for the local communities and also sustain small populations of the endangered Ethiopian wolf (*Canis simensis*). Questionnaires were designed to assess ecological and socio-economic characteristics of the livelihoods of the Amhara people living in Mount Abune Yosef and their attitudes toward Afroalpine and Ethiopian wolf conservation. Of the 120 households interviewed, selected randomly from across eight villages, 80 % benefited from natural resources by grazing their livestock and harvesting firewood and grasses. The majority (90 %) also suffered from livestock predation by Ethiopian wolves and common jackals *(Canis aureus)* and crop raiding by geladas (*Theropithecus gelada*), birds, and rodents, yet more than half reported a positive attitudes toward Ethiopian wolves (66 %). People with positive attitudes tended to live close to the communal land, to own more livestock, and to be unaffected by conflict. Many also recognized the need to protect the Afroalpine habitats of Abune Yosef (71 %), and this attitude predominated among the literate, households that owned land, had smaller herds and were further away. We discussed how people’s attitudes were modulated by human-wildlife conflicts and by the benefits derived from the access to natural resources in communal land, and the implications for the conservation of Afroalpine ecosystem and the flagship Ethiopian wolf.

## Introduction

People who perceive economic benefits and enjoy unrestricted access to natural resources are expected to be supportive of ecosystem conservation efforts (Kellert 1985; Brüner et al. 2001; Walpole and Goodwin 2001; Wang and Macdonald 2006). However, if the economic consequences of human-wildlife conflict for local poor households become unbearable, attitudes toward the conservation of biodiversity can change significantly (Oli et al. 1994; Treves and Karanth 2003; Naughton-Treves et al. 2005; Thirgood et al. 2005; Woodroffe et al. 2005; Treves 2007).

In the highlands of Ethiopia, the traditional livelihoods of the Amhara people combine subsistence agriculture with livestock rearing, complemented by access to natural resources in communal Afroalpine areas, including water, construction materials, firewood, and grazing land (Gebremedhin and Swinton 2002; Ashenafi et al. 2012). The Afroalpine ecosystems of Ethiopia have been used for millennia under unrestricted access by the surrounding communities (Ashenafi and Leader-Williams 2005; Ashenafi et al. 2012), but the rapidly growing human populations are posing new challenges. The intensification of farming and livestock grazing is resulting in environmental degradation and conflicts with wildlife across Ethiopia (Stephens et al. 2001; Bekalo and Bangay 2002; Yirga et al. 2012), with potential consequences for the conservation of Afroalpine ecosystems (Ashenafi and Leader-Williams 2005; Marino 2003).

The Ethiopian highlands harbor an exceptionally diverse array of endemic species, among them the endangered Ethiopian wolf (*Canis simensis*) (Marino and Sillero-Zubiri 2011), gelada baboon (*Theropithecus gelada*), walia ibex (*Capra walia*), and several species of *Rhizomyidae* and *Murinae* rodents (Yalden and Largen 1992). The communal land in Mount Abune Yosef, North Wollo, is a good example of a high-biodiversity Afroalpine remnant which is critically important for the Amhara people and for Ethiopian wolves alike (Ash 2001; Marino 2003). In such a setting, people’s attitudes toward conservation can have important consequences for the survival of wolves and other highland endemics, and for the long-term sustainability of traditional livelihoods. In particular, it is likely that communities develop negative attitudes toward wildlife conservation as a result of livestock predation or crop raiding (Sekhar 1998; Treves 2007; Marino 2003).

Previous studies of human-wildlife conflict in Ethiopia have been conducted within protected areas (Yihune et al. 2008, 2009; Tessema et al. 2010), where contacts between people and wildlife are largely restricted to the protected area boundaries. In densely populated highlands of Mount Abune Yosef in Wollo, however, these interactions will be more frequent and leading to conflicts due to livestock predation by common jackals (*Canis aureus*) and Ethiopian wolves (Marino 2003; Marino et al. 2010) and damage to barley fields caused by geladas and rodents (Dunbar 1998; Yihune et al. 2009; Kifle et al. 2013). If the economic consequences of these conflicts are significant for the local farmers, negative attitudes toward conservation might arise (Treves and Karanth 2003; Yirga et al. 2012; Winterbach et al. 2013). To test this hypothesis, we conducted semi-structured interviews to generate qualitative and quantitative information about local livelihoods and wildlife conflicts in Mount Abune Yosef, taking into account the benefits derived from access to natural resources in communal land and how these affect people’s attitudes and tolerance toward wildlife.

## Methods

### Study Area

**Fig. 1:**
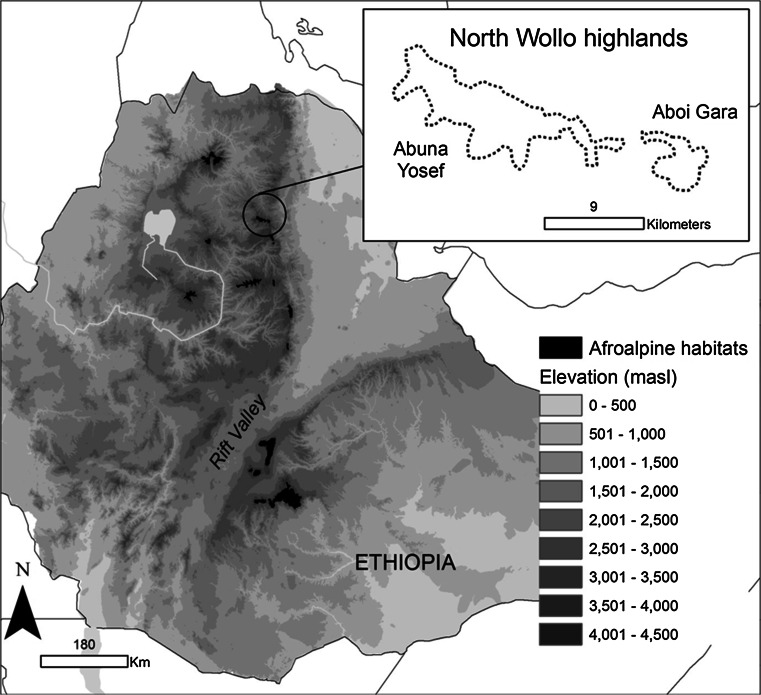
Map indicating areas of Afroalpine habitats in the Ethiopian highlands. Inset shows study area of Abune Yosef and adjacent Aboi Gara

Mount Abune Yosef (hereafter AY) is located in the Lasta district of North Wollo Zone, between 12°8′7″N and 39°15′7″E (Fig. 1). This isolated mountain reaches up to 4286 m a.s.l and contains approximately 50 km^2^ of suitable wolf habitats (Marino 2003; Saavedra 2009). The climate is moist and cold, with a wet season from June to October, and a dry season from November to May. The average annual rainfall is 2,000 mm and the mean annual temperature ranges between 7.5 and 11 °C (ESP 2001). The highlands of North Wollo are watersheds for three main river basins (Tekeze, Awash, and Blue Nile basins). The Amhara people settled in these highlands for millennia and still use traditional methods for farming and bring their livestock to graze in Afroalpine pastures. AY also has cultural value due to its scenery and the presence of endemic animals, which attract a growing number of visitors, and to centuries as an important religious site, with many churches and monasteries (Saavedra 2009). Mount Abune Yosef is located close to the holy city of Lalibela, one of Ethiopia’s top tourist attractions. Taking advantage of this situation, a community-based tourism initiative was started a few years ago, with support of international NGOs.

The landscape is open and dominated by grasslands and heathlands, with steep slopes covered by rock and shallow soils, and valleys and depressions, with deep black soils, sustaining an important green biomass. The mosaic of Afroalpine vegetation types includes ‘*guassa’* grasslands (*Festuca* spp.), giant lobelias (*Lobelia rhyncopetalum*), *Euryops* bushes locally known as ‘*chifra*’, ‘*kirshiba,*’ or ‘*charranfe*’, and remnant patches of *Erica* spp. forests. In AY, there are 43 species of mammals, including seven Ethiopian endemics, and 221 species of birds, of which 16 are endemic, making it the second most important bird area in the country (EWNHS 1996; Lepage 2006; Saavedra 2009).

### Data Collection and Analysis

Pilot surveys were initially conducted in 16 households to gather background information and to test and adapt the questionnaire. Between October 2009 and April 2011, 120 households were interviewed, selected randomly from a list of 2014 households across eight villages in four Peasant Associations or ‘*kebeles*’ (the smallest local administration unit). The inhabitants of these villages visit the Afroalpine area of AY frequently to herd their livestock, to collect grass and firewood, or en route to local markets. It was agreed that the information collated will only be used for the purpose of the study.

The questionnaire (Appendix) was designed to evaluate the local uses of Afroalpine natural resources for own consumption or to commercialize (e.g., firewood and grasses are sold for cash or exchanged in local markets to compensate for goods and services that are not locally unavailable or in deficit), the extent to which households were affected by predation, and their views regarding the need to protect Abune Yosef, and their attitudes toward wolf. We collected information about the heads of the household that is expected to influence attitudes, such as sex, marital status, family size, and educational status, together with others related to their livelihoods, expected to influence as well their degree of dependence upon additional Afroalpine resources, such as firewood and grasses, to compensate their livelihoods; namely whether the family owns land for agriculture (‘own land’), the size of the plot (‘land size’), whether they keep livestock (‘herd size’ as number of heads) and the grazing regime (months grazing at Mount Abune Yosef, and by season: dry and wet). Regarding conflicts with wildlife, the heads of households were asked about the type of conflict they are exposed to, namely livestock predation and crop raiding, and their frequencies and overall trends. Regarding attitudes, people were asked their view about the need to protect AY and their attitudes toward wolf and possibility that wolves and people co-exist in AY.

To calculate the financial benefits derived from the commercialization of natural resources, local market prices for the year 2010/11 were considered (load of firewood = 20 Ethiopian birr; load of hay & thatch grass = 50 Ethiopian birr), and converted to USA dollars at an exchange rate of 1 US$ = 10 Ethiopian birr.

Descriptive statistics were used to describe local livelihoods, and cross tabulations and Chi square tests for categorical variables. We used logistic regressions to explore variations in peoples’ attitudes, considered as binary response (e.g., yes/no answers: 0 for a negative response and 1 for positive). All analyses were conducted with the statistical packages SPSS (version 16) and SAS-JMP 5 software.

## Results

### Socio-economic Characteristics and Resource Uses

**Fig. 2:**
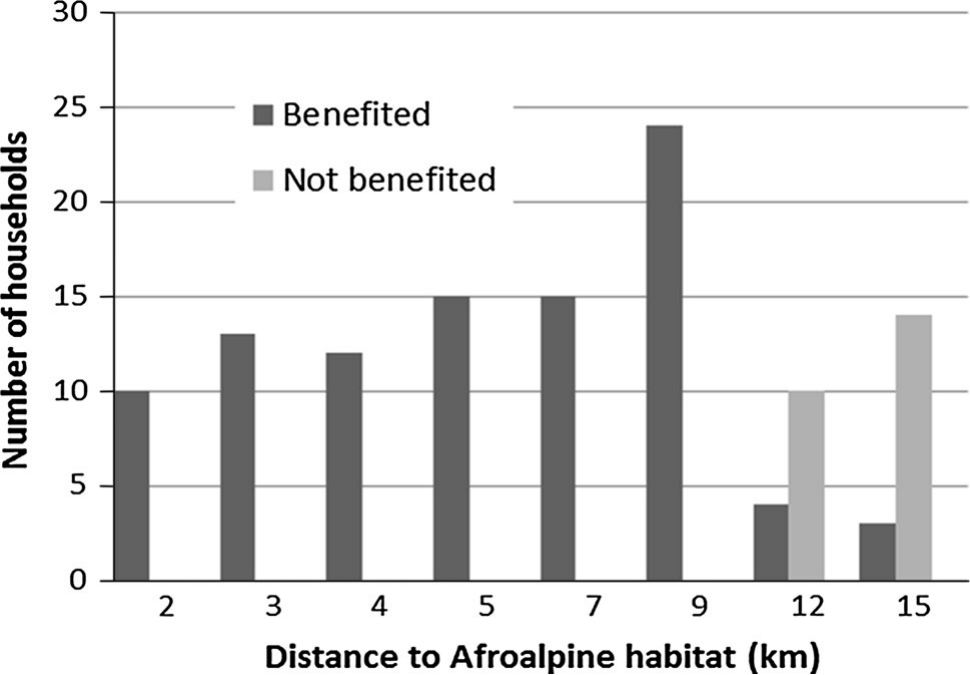
Number of households that benefited from using natural resources at various distances from Abune Yosef

Regarding the socio-economic profile, most heads of household were married men and over half of them illiterate. The majority owned land (average 0.7 ha) and a small herd of livestock (average 14 heads) (Table 1). Eighty percent of the households benefited economically from the use of natural resources, but this proportion varied across villages (Table 2) and over half (61.7 %) used Afroalpine pastures to graze, for at least 9 months a year. All households located within 10 km of Mount Abune Yosef exploited some natural resource, but only a small proportion of the household located further away (Fig. 2). More landless households utilized natural resources in comparison with the households that owned agricultural land (*X*^*2*^ = 4.62, df = 1, *P* < 0.05).

**Table 1:** Characteristics of the 120 households interviewed

	Number	Percentage of households (%)
Sex		
Male	103	85.8
Female	17	14.2
Marital status		
Married	103	85.8
Single	17	14.2
Educational status		
Illiterate	63	52.5
Literate	57	47.5
Own land		
Yes	104	86.7
No	16	13.3
Affected by wildlife damage		
Affected	108	90.0
Not affected	12	10.0
AY needs protection		
Yes	85	70.8
No	35	29.2
Responsible to protect AY		
Community	96	80
Government	24	20
Attitude toward Ethiopian wolf		
Positive	79	65.8
Negative	41	34.2
Can co-exist with wolves		
Yes	72	60
No	48	40

Continuous variables	Minimum	Maximum	Mean	SD
Age	18	80	47.5	12.268
Family size	1	10	5.7	1.827
Distance to Afroalpine area (km)	2	15	7.72	4.199
Herd size (number of heads)	0	78	13.69	10.509
Land size (ha)	0	2.3	0.70	0.440

**Table 2:** Households per village that utilize natural resources from Abune Yosef

Village	Households sampled	Benefit from natural resource uses
Eyebelay	17	13 (76.5 %)
Korit	11	11 (100 %)
Abune Yoseph	14	14 (100 %)
Latige	10	10 (100 %)
Kassegne	22	20 (90.9 %)
Enjafat	13	4 (30.8 %)
Shegla	11	9 (81.8 %)
Ybaro	22	15 (68.2 %)
Total	120	96 (80 %)

Regarding the uses of natural resources, two thirds of the households reported collecting firewood, and many harvested thatching grass and hay (Table 3). Other natural resources were used as farming implements, construction materials, and medicinal plants, while tourism provided income to nearly one third of the households interviewed (Table 3).

**Table 3:** Types of economic resources/services households obtained from Abune Yosef (*n* = 120)

Resources	Number of households	%
Firewood	89	74.2
Thatching grass	76	63.3
Hay grass	66	55
Tourism	35	29.2
Other	92	76.7

The estimated financial benefit perceived by households was on average US$ 92 per year, ranging from US$ 5 from firewood sales to US$ 300 from tourism revenues (tourist guiding, renting pack animals, and selling locally made items) (Table 4).

**Table 4:** Economic benefits that households perceived from the use of natural resources in Abune Yosef

Resource	Annual income (in Ethiopian birr)				*N*
	Minimum	Maximum	Mean	SD	
Firewood	50	1050	324.8	272.8	89
Thatching grass	75	1500	477.4	362.8	76
Hay grass	120	1500	421.6	303.6	66
Tourism	100	3000	659.1	659.1	35

### Conflicts and Attitudes

Nearly every household surveyed reported some form of wildlife damage, including livestock predation by Ethiopian wolves and common jackals, and crop damage by geladas, birds, and rodents (Table 5). Half of the households suffered both from livestock predation and crop raiding. The type of conflict differed across the villages (*P* < 0.01), while people in *Enjafat* and *Latgie* did not report wildlife-related damage, most households in *Eyebelay*, *Korit*, and *Ybaro* experienced both livestock predation and crop raiding (Table 5).

**Table 5:** Wildlife conflicts and percentage of households affected across villages

Villages	Households sampled	With conflicts	%	Livestock predation only	Crop damage only	Both
Eyebelay	17	17	100	2 (11.8 %)	3 (17.6 %)	12 (70.6 %)
Korit	11	11	100	3 (27.3 %)	1 (9.1 %)	7 (63.6 %)
Abune Yoseph	14	13	93	6 (42.9 %)	3 (21.4 %)	4 (28.6 %)
Latgie	10	7	70	6 (60 %)	0	1 (10 %)
Kassegne	22	20	91	9 (40.9 %)	1 (4.5 %)	10 (45.5 %)
Enjafat	13	9	69	3 (23 %)	2 (15.4 %)	4 (30.8 %)
Shegla	11	9	82	3 (27.3 %)	0	6 (54.5 %)
Ybaro	22	22	100	5 (22.7 %)	1 (4.6 %)	16 (72.7)
Total	120	108	90	37 (30.8 %)	11(9.2 %)	60 (50 %)

Most people believed that Ethiopian wolf numbers were decreasing in AY, alleging habitat loss, emigration, and competition with jackals as the causes (43, 26, and 21 % respectively). However, people reported seeing Ethiopian wolves on average 6.0 ± 0.7 times over the previous 12 months, compared with 13.0 ± 1.7 per year when they were asked over the last 5 years. Wolves were seen alone or in groups of up to 12 (average 4.2 ± 0.2 wolves), and most commonly in the early and late hours of the day (59 % at dawn and dusk, the rest at dawn only).

In spite of the relatively high frequency of livestock predation reported (affecting more than 90 % of the households), more than half of people believed that people and Ethiopian wolves can co-exist in AY (60 %) and that they feel positively about them (66 %). This positive attitude was most common among households located close to Afroalpine areas, households with larger herds, and those less affected by wildlife predation (Table 6).

**Table 6:** Result of logistic regression explaining people’s attitudes toward Ethiopian wolves (1 = positive. 0 = negative)

Explanatory variables	Estimate	Std Error	Chi Square	*P* value
Age	0.005	0.024	0.044	0.838
Marital status (1 = married)	−1.522	0.952	2.555	0.110
Sex (1 if male)	1.252	0.944	1.762	0.184
Family size	0.280	0.150	3.486	0.062
Educational status (1 = literate)	0.657	0.567	1.345	0.246
Distance to Afroalpine area	−0.141	0.057	6.111	0.013
Own land (1 if yes)	0.154	0.801	0.037	0.848
Herd size	0.132	0.044	9.128	0.003
Affected by wildlife damage (1 = not affected)	2.193	0.742	8.744	0.003
*R* ^2^	0.361			
Correctly predicted percentage	76.7			
Observations	120			

The majority of the respondents (71 %) perceived a need to protect Abune Yosef, and many preferred a community-based approach (80 %) over a government-led one. Interestingly, whether people used natural resources or not, whether they were affected by conflict or not, did not affect their perception. The households that considered some form of conservation were necessary tended to have literate heads, to own land, to have smaller herds, and to live further away from the Afroalpine area (Table 7).

**Table 7:** Result of logistic regression explaining people’s perception of the need to protect the Afroalpine ecosystem (1 = there is a need to protect AY, 0 = there is not)

Explanatory variables	Estimate	Std error	Chi square	*P* value
Age	−0.008	0.035	0.051	0.821
Marital status (1 = married)	0.753	1.113	0.457	0.499
Sex (1 = male)	−0.833	1.110	0.562	0.453
Family size	0.223	0.175	1.611	0.204
Educational status (1 = literate)	2.723	0.729	13.938	0.000
Time living at AY	0.017	0.024	0.516	0.473
Distance to Afroalpine area	0.248	0.096	6.601	0.010
Own land (1 = yes)	2.593	0.928	7.812	0.005
Herd size	−0.087	0.037	5.615	0.018
Firewood collection (1 = yes)	0.085	0.991	0.007	0.932
Thatching grass collection (1 = yes)	0.144	0.868	0.028	0.868
Hay grass collection (1 = yes)	0.490	0.786	0.390	0.533
Affected by wildlife damage (1 = not affected)	0.625	0.873	0.513	0.474
*R* ^2^	0.456			
Correctly predicted percentage	78.3			
Observation	120			

## Discussion

Our study exemplifies how people’s attitudes toward conservation and wildlife can be modulated by socio- economic characteristic and by conflicts with wildlife, in a case where open resource uses might conflict with the conservation of a charismatic endemic such as the Ethiopian wolf.

The local communities of Abune Yosef resembled other rural communities that engage in small-scale agriculture and livestock rearing, and which depend on biodiversity for their subsistence, for example, as a source of energy, building materials, drinking water, and products that can be bartered and sold in local markets to access goods and services that are not locally available (Lewis et al. 1990; Newmark et al. 1993; Winterbach et al. 2013). Subsistence farmers around AY exploited diversified goods and environmental services from the Afroalpine ecosystem, as do communities in other Afroalpine areas of Ethiopia under some level of resource use management such as the Guassa Conservation Area (Ashenafi 2001) and the Simien Mountains National Park (Yihune et al. 2008). The communities of AY use Afroalpine pastures intensively, in many cases all year round, and depend on Afroalpine bushes as sources of firewood and to commercialize. This is the reality across rural Ethiopia, where most people depend on firewood for cooking and lighting, and on livestock as a form of financial insurance for times of necessity (Taddese 2001). The communities of AY also benefited financially by the commercialization of *Festuca* grasses and hay, used as fodder and for thatching and basket making (Ashenafi et al. 2012; Jacob et al. 2014), and other wild plants used for medicine and construction (Ashenafi 2001). Interestingly, a considerable proportion of the households benefited from tourism, revealing a significant financial impact of this touristic activities led by the local communities of AY. This adds to the evidence that tourism can be an important alternative source of income when developed as a community-based initiative, like in Guassa Community Conservation Area in Ethiopia (Ashenafi and Leader-Williams 2005) and in other African countries (Binns and Nel 2002; Hutton and Leader-Williams 2003; Lindsey et al. 2007; Hoole 2009; Mbaiwa and Stronza 2010). The estimated annual income derived from ecosystem goods and services provided by Mount Abune Yosef was US$ 92 per household, a substantial economic contribution considering that the per capita average Gross National Income (GNI) of Ethiopians was US$ 470 in 2013 (World Bank 2013).

Consequently, most people considered the protection of natural resources positively, as their livelihoods will logically depend on the long-term persistence of these ecosystem services. Their attitudes, however, vary with socio-economic factors as in other rural areas of Africa, including the benefits derived from environmental goods and ecosystem services as well as the economic losses due to livestock predation (e.g., Romanach et al. 2007; Lagendijk and Gusset 2008; Dickman 2010). As expected, literacy was associated to people’s perceptions of the need for conservation, a common pattern globally in North America (Kellert et al. 1996), South Africa (Lagendijk and Gusset 2008), and Uganda (Kugonza et al. 2009). Farmers that own land, and thus have a rural land certification provided by the local Land Administration Office, have right to use the farmland and surrounding natural resources, and thus more self-assured to benefit from conservation. In comparison, landless households exploit natural resources in unregulated ways, and might therefore feel threatened by conservation initiatives. This coincides with studies in Ethiopia and elsewhere showing that farmers that own land are more collaborative toward biodiversity conservation activities, than farmers using state owned or non-private land (Ellis 1996; Rahmato 2003; Teklu 2003; Romanach et al. 2007; Kugonza et al. 2009). Also the people living further away from the communal land and with smaller herds recognized more the need to protect Abune Yosef. One logical explanation is that the families living close to the communal land and with more heads of livestock are exerting competition upon them, so they perceive a greater urgency to protect the resources in the longer term and for the benefit of everyone. Among the other group of families, some might perceive conservation as a threat, as this entails restrictions to harvesting, traditional free grazing rights, and displacement.

With respect to the charismatic Ethiopian wolf, a flagship for the conservation of Afroalpine habitats, most people were positive about the wolves and believed on human-wolf co-existence. However, the challenges that livestock predation might have upon the local households economies were evident in the association between negative attitudes, smaller herds (i.e., households that will face a relatively high economic costs), and past exposition to livestock predation. Still, persecution and retaliatory killings were never reported as a cause for the perceived decline in the wolf population, and are not considered as threats to Ethiopian wolves elsewhere (Sillero-Zubiri and Macdonald 1997; Ashenafi et al. 2005; Marino et al. 2013). These contradicts with lessons from many other regions of the world, where predation by wild carnivores almost invariably generates negative attitudes among rural residents, and the ensued retaliation leading sometimes to severe population declines (e.g., Woodroffe 2000; Bauer 2003; Sogbohossou et al. 2011).

## Conservation Implications

Our study describes the delicate equilibrium between the socio-economic needs of local people and the need to protect the Afroalpine ecosystem in AY, because local livelihoods not only depend on the income generated from natural resources but also suffered from wildlife-related costs. Careful management will be required if the dual goals of wildlife conservation and economic livelihood for communities are to be met (Linnell et al. 2001; Hutton and Leader-Williams 2003; Winterbach et al. 2013).

Although Ethiopian wolves are specialized rodent hunters, this study shows that in the heavily populated highlands of North Ethiopia they are common predators of livestock, possibly a reflection of dietary adjustments to less abundant rodent prey and high livestock availability (Sillero-Zubiri and Gottelli 1995; Marino et al. 2010). Still, due to their high charisma, conflicts have been kept on check. A reason for concern is the possibility of conflicts increasing, as human and livestock populations in rural Ethiopia continue to grow, threatening the sustainability of the local livelihoods and the emergence of retaliation (Dovie et al. 2006; Lagendijk and Gusset 2008). Understanding and mitigating the risk of livestock predation should be considered a priority for AY and other Ethiopian wolf populations. Results from the Simien Mountains National Park (Yihune et al. 2008) and other protected areas of Africa (e.g., in Cameroon by Van Bommel et al. 2007 and in Botswana by Schiess-Meier et al. 2007) indicate that predation will be highest close to the Afroalpine habitats where jackals and wolves live, and that predation will vary with the prevailing grazing regimes and guarding techniques.

Interventions designed to ensure access to natural resources while promoting long-term sustainability will contribute to maintain positive attitudes among people in AY (Dickman 2010; Winterbach et al. 2013), and continued willingness to co-existence with carnivores (Kellert et al. 1996; Hutton and Leader-Williams 2003; Bath et al. 2008; Dickman 2010). Considering that, demand for land in itself is a major threat to the conservation of Afroalpine ecosystems, opportunities for alternative incomes should always be promoted to ensure positive attitudes toward conservation among landless households in AY, of which tourism is a good example.
